# Investigating the Moderating Effect of Language Attitude in the Interplay Among Social Media Addiction, Social Pain and Internet Trolling in College Students

**DOI:** 10.3390/bs15050586

**Published:** 2025-04-27

**Authors:** Qingshu Xu

**Affiliations:** 1 School of Aeronautics, Shandong Jiaotong University, Jinan 250357, China; qsxu1987@umd.edu; 2 College of Education, University of Maryland, College Park, MD 20742, USA

**Keywords:** social media addiction, social pain, internet trolling, language attitude

## Abstract

This study investigates the moderating effect of language attitude on the relationships among social media addiction, social pain, and internet trolling among college students. A sample of 891 students from various colleges and universities completed validated measures assessing their levels of social media addiction, social pain, internet trolling, and language attitude. Using a latent variable approach within a multigroup structural equation modeling (SEM) framework, participants were divided into three groups (high, medium, and low language attitude) based on their language attitude scores. The SEM analysis revealed distinct patterns across groups. In the high language attitude group, both social media addiction and social pain significantly predicted internet trolling, with standardized regression coefficients of 0.564 and 0.728, respectively. In the medium language attitude group, the predictive effects remained significant; however, the magnitude of the coefficients decreased markedly (0.264 for social media addiction and 0.562 for social pain). In contrast, in the low language attitude group, neither social media addiction nor social pain emerged as significant predictors of internet trolling. Interestingly, the covariance between social media addiction and social pain remained consistent across the three groups, suggesting a stable interrelationship irrespective of language attitude level. These findings imply that language attitude plays a crucial moderating role in the interplay among social media addiction, social pain, and internet trolling. Specifically, higher levels of language attitude appear to amplify the effects of social media addiction and social pain on internet trolling behavior, while lower levels attenuate these associations. The results underscore the importance of considering individual differences in language attitudes when developing intervention strategies aimed at mitigating problematic online behaviors among college students.

## 1. Introduction

In today’s hyper-connected world, social media platforms have revolutionized the way individuals interact, share information, and construct their identities (Abdulsalim et al., 2025; Li et al., 2025; Yang et al., 2025). However, the pervasive use of these platforms has also been linked to a range of adverse psychological and behavioral outcomes (Thibault, 2025; Yang et al., 2025; Yoosefi et al., 2025). Among these, social media addiction has emerged as a critical concern, often manifesting in compulsive online behaviors that not only disrupt offline functioning but also foster negative interpersonal dynamics (Yoosefi et al., 2025). One such detrimental behavior is internet trolling—a phenomenon characterized by deliberately provocative or hostile online interactions—which can escalate into forms of cyber bullying (March et al., 2024; Spencer, 2025). Recent studies suggest that the relationship between social media addiction and trolling behavior is not straightforward; instead, it appears to be influenced by individual differences in language attitude and tolerance toward offensive language (Bishop, 2015a).

Language attitude—the degree to which individuals perceive and react to potentially offensive or aggressive language—plays a pivotal role in shaping online interactions (Kirilenko, 2024). Preliminary evidence indicates that individuals with heightened sensitivity may respond more intensely to provocative language via emotional channels or behavioral channels, potentially altering the impact of social media addiction on trolling behaviors. Moreover, language tolerance—the ability to endure or accept offensive language without excessive emotional reactivity—may mitigate or amplify this relationship (Soukup, 2012). In parallel, social pain, defined as the emotional distress resulting from perceived social exclusion or rejection, has been identified as both a correlate of social media addiction and a potential antecedent of trolling behavior (Kim et al., 2025). Notably, the strong correlation between social media addiction and social pain suggests that individuals who are excessively engaged online may be more vulnerable to feelings of social distress, which, in turn, may predispose them to engage in trolling or cyber bullying behaviors (Cho et al., 2025).

College students are especially prone to be influenced by all factors listed in the previous part, namely, social media addiction, social pain and internet trolling (Abdulsalim et al., 2025). The present study seeks to investigate these interrelated dynamics in the population of college students using a structural equation modeling (SEM) approach. Specifically, we examine the causal relationship between social media addiction and internet trolling, and explore how this association is moderated by offensive language attitude and tolerance. Furthermore, we assess the correlation between social media addiction and social pain, and test whether social pain directly contributes to trolling behavior. Finally, we investigate if the causal link from social pain to cyber bullying is similarly moderated by language attitude and tolerance. By integrating these constructs into a comprehensive model, this study aims to fill existing gaps in the literature and offer nuanced insights into how individual differences in language processing may buffer or exacerbate the negative outcomes associated with problematic social media use.

Understanding these complex relationships is crucial for developing targeted interventions and policies that not only address social media addiction but also mitigate the broader social and psychological harms of online aggression.

## 2. Literature Review

### 2.1. Social Media Addiction

Social media addiction is increasingly regarded as a specific manifestation of the broader construct of Internet addiction (Kakulte, 2025). Whereas early research on Internet addiction focused on diverse online activities, accumulating evidence suggests that individuals are not addicted to the Internet per se but to particular online behaviors. Notably, among adolescents, problematic use is most convincingly linked to two specific activities: gaming and social media use (Zewude et al., 2025). As such, social media addiction is conceptualized as a subtype of Internet addiction, sharing many of its core features and diagnostic criteria—as exemplified by the adaptation of the nine DSM-5 criteria for Internet Gaming Disorder (IGD) to form the basis of the Social Media Disorder (SMD) Scale (van den Eijnden et al., 2016).

Social media addiction is defined as a compulsive, excessive, and maladaptive engagement with social networking platforms (e.g., Facebook, Instagram, TikTok) that interferes with daily functioning (Lorenzo-Rumbo et al., 2025; Sönmez Sari et al., 2025). It is characterized by symptoms analogous to substance-related addictions, including preoccupation with social media, tolerance (i.e., the need to increase usage to achieve the same satisfaction), withdrawal symptoms when access is denied, and continued use despite adverse consequences (van den Eijnden et al., 2016). Although the Diagnostic and Statistical Manual of Mental Disorders (DSM-5) has not yet formally recognized social media addiction, there is a growing consensus in the literature that such pathological use represents an emerging mental health issue—especially among adolescent smartphone users (Abdulsalim et al., 2025; Yang et al., 2025).

The literature suggests that several factors may contribute to the development of social media addiction. First, the inherent design of social media platforms plays a significant role. Features such as endless scrolling, real-time notifications, and variable reinforcement through likes and comments create a reward system that can foster compulsive use (van den Eijnden et al., 2016). Second, psychological factors—including low self-esteem, social anxiety, and a strong need for social validation—make certain individuals more vulnerable to overuse these platforms (Kagan et al., 2025). The pervasive culture of social comparison, where users constantly measure their self-worth against idealized representations of others, further exacerbates this vulnerability. Finally, environmental and situational factors, such as peer pressure and the increasing normalization of constant online connectivity, also contribute to the risk of developing addictive behaviors (Piko et al., 2025).

The consequences of social media addiction are multifaceted and primarily concern adverse mental and psychosocial outcomes. Compulsive use has been associated with increased levels of depression, anxiety, and loneliness, partly due to the experience of social pain—emotional distress stemming from perceived rejection or isolation (Wang et al., 2024). Moreover, excessive engagement with social media can disrupt sleep patterns, reduce attention span, and impair academic and occupational performance (Abdulsalim et al., 2025; Lorenzo-Rumbo et al., 2025). Social media addiction may also strain interpersonal relationships, as time spent online detracts from face-to-face interactions and can lead to conflicts with family and peers (Qi et al., 2024). While some users may initially benefit from the social support provided by these platforms, chronic overuse tends to yield a net negative impact on overall well-being (Liao et al., 2025). Many individuals who have no outlet to release their stress may resort to internet trolling as a means of alleviating their pain and suffering (Ao & Mak, 2021; Bishop, 2015a).

### 2.2. Social Pain

Social pain is understood as the negative emotional experience that arises when one perceives social exclusion, rejection, or relational devaluation (Sullivan & de C Williams, 2025). Social pain is not merely about the observable act of exclusion—instead, it captures the internal “hurt” felt by the individual, distinguishing it from other negative emotions like shame or guilt. This concept reflects a subjective, affective response to being left out or devalued in interpersonal contexts, whether in romantic relationships, friendships, or broader social groups (Stangier et al., 2021).

The construct of social pain has been linked to theories suggesting an overlap between the neural processing of physical and social pain (Schwarz et al., 2021). In other words, the brain regions activated during experiences of social rejection share similarities with those involved in the sensation of physical pain (Sullivan & de C Williams, 2025). This overlap highlights the evolutionary significance of social bonds. Just as physical injury can jeopardize survival, social rejection can threaten our fundamental need to belong. In fact, social pain encapsulates the deep-seated emotional hurt that stems from perceived social disconnection, a response that is as critical to our well-being as it is rooted in our neurobiology and evolutionary history (Stangier et al., 2021; Sullivan & de C Williams, 2025).

With the rapid acceleration of social mobility and increasingly diverse modes of interpersonal interaction, individuals are facing ever-greater challenges in their social relationships. Negative emotional experiences that arise when social bonds are threatened or social value is devalued—such as through social exclusion, peer rejection, or the loss of close ones. These negative emotional experiences have become a major focus in psychological research due to their detrimental effects on mental health (Kunstman et al., 2025).

Severe social pain might lead some of the suffered to smartphone use addiction or social media addiction (Wang et al., 2024) and others to internet trolling (Buckels et al., 2019) to mitigate.

### 2.3. Internet Trolling

With the rise in social media, internet trolling has become a widespread phenomenon across cultures worldwide (Türk Kurtça & Demirci, 2023). In essence, trolling involves deliberately posting insulting or provocative content online with the goal of sparking heated arguments or conflicts. Such behavior not only generates negative feelings—including hatred, humiliation, despair, and loneliness—but also heightens the risk of suicidal thoughts and self-harm (Coles & West, 2016). Moreover, trolling can diminish the credibility of information sources by fueling the rapid spread of negative commentary, which in turn creates an increasingly hostile digital environment (Dynel, 2016). Internet trolling is defined as the act of engaging in deceptive, disruptive, and provocative online communication for self-satisfaction—specifically to elicit negative emotional reactions from others (Buckels et al., 2019; Buckels et al., 2014).

Although trolling shares certain features with other antisocial online behaviors such as cyberbullying and flaming, it is fundamentally different in terms of its form, content, intent, and outcomes—differences that have not been thoroughly investigated (Coles & West, 2016). For instance, while trolling can be viewed as a subset of cyberbullying given that both involve attacking others, trolling is more centered on deception and causing senseless disruption. In contrast, cyberbullying typically involves individuals with a clear social identity and explicit intent to harm. Similarly, whereas trolling often targets vulnerable or marginalized groups, flaming is aimed at provoking an entire online community into a heated debate (Atsariyasing et al., 2025).

Furthermore, research has highlighted that both individual traits and environmental factors play key roles in the emergence of trolling behavior (March et al., 2024; Türk Kurtça & Demirci, 2023). Studies indicate that those who are suffering from social pain are generally more prone to engage in trolling and that younger individuals tend to troll more frequently than their older counterparts (Buckels et al., 2019). Additionally, among the spectrum of dark personality traits, a propensity for sadism is particularly strongly associated with trolling (Buckels et al., 2019). Factors such as online anonymity and the prevalence of uncivil posting further encourage such behavior (Bishop, 2014).

### 2.4. Language Attitude

Language attitude has evolved into a key concept within sociolinguistics since the pioneering studies of Gardner and Lambert (1972). These foundational works established that individuals’ evaluations of a language are not only reflections of cultural and social biases but also predictive of subsequent language-related behaviors (Ryan & Giles, 1982). Over time, language attitude has become central to understanding how people perceive and use language.

Most contemporary definitions of language attitude draw on psychological theories of attitude and generally share two main features (Xie & Peltokorpi, 2025). First, research typically focuses on a relatively uniform subject—language attitude is often defined as an individual’s evaluative stance toward a particular language or its variations. For example, definitions in the literature describe language attitudes as feelings toward a whole language (e.g., Hebrew), its specific features (e.g., a given phonological variant), its functional use (e.g., Hebrew for secular purposes), or even its role as a marker of group identity (Rosiak, 2025). Second, there is diversity in how researchers divide the dimensions of language attitude. Some adopt a dualistic approach that distinguishes between, for instance, the language’s perceived value and the behavioral tendency it elicits (Xie & Peltokorpi, 2025), while others favor a pioneering model that further separates the evaluative process into appreciation, judgment, and affect (Soukup, 2012).

Although the predominant focus has been on macro-level language attitudes—those directed toward entire languages or major variants—recent work has highlighted the need to explore more nuanced, micro-level attitudes as well (Soukup, 2012). This perspective has led some scholars to redefine language attitude using the triad theory of social psychological attitude. Under this approach, language attitude is seen as a structured, relatively stable internal state comprising three components: cognition, affect, and behavioral tendency. This redefinition not only captures broad evaluations of language but also accommodates more specific attitudes, such as those toward particular sets of words (e.g., sexual swear words), which, in turn, influence behavior (Wei & Chen, 2021).

Empirical research consistently supports a strong relationship between language attitude and behavior. Gardner’s socio-educational model clearly demonstrates that positive attitudes toward a language are associated with increased motivation, higher engagement, and more frequent language use (Gardner & Lambert, 1972). Similarly, Ryan and Giles (1982) found that favorable language attitudes lead to greater willingness to use the language in intergroup interactions and various social contexts. Such findings have been extended by later studies—for example, research examining the ATOL-M scale has shown that individuals’ attitudes toward offensive language in media can predict their behavioral responses and enjoyment of media content (Shafer & Kaye, 2015). These studies collectively indicate that language attitude not only reflects evaluative judgments but also translates directly into observable linguistic behaviors, such as language choice, code-switching, and even participation in language-based social interactions (Bednarek, 2019; Williams & Uebel, 2021).

### 2.5. Hypothesis

**Figure 1:**
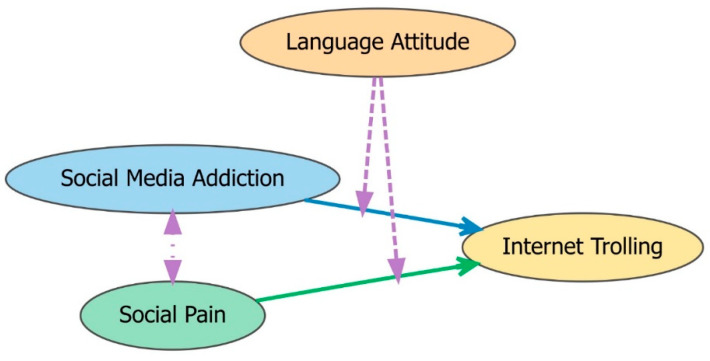
Hypothetical Model Structure.

Drawing on previous research that has linked negative social experiences and heightened online behaviors, as well as studies examining the role of language attitude in shaping communicative responses, we propose the following hypotheses (see Figure 1):

**H1:**  *Individuals experiencing higher levels of social pain are more likely to engage in internet trolling*.

**H2:**  *Social media addiction significantly predicts internet trolling behavior*.

**H3:**  *Social media addiction is strongly positively correlated with social pain*.

**H4:**  *Language attitude moderates the relationships outlined in H1 and H2*.

Together, these hypotheses aim to elucidate the interplay among social pain, social media addiction, and internet trolling, while also considering the moderating influence of language attitude on these relationships.

## 3. Method

### 3.1. Design and Procedure

This study employed a cross-sectional survey design to examine the interplay among social media addiction, social pain, and internet trolling, with language attitude serving as a moderating variable. Data were collected through an online questionnaire hosted on a secure survey platform. Participants were first provided with an overview of the study, including its purpose, confidentiality assurances, and the voluntary nature of participation. After consenting, respondents completed a battery of self-report measures comprising 42 items, which took approximately 25–40 min. Upon completion, participants were debriefed and provided with resources for psychological support if needed.

### 3.2. Setting

This cross-sectional survey was conducted online, starting from 11 October 2024 and ending on 20 December 2024. Using snowball sampling approach, 1545 unique invitations were distributed via university’s learning-management system. Ethical approval was granted and all respondents provided informed consent online. We received 1240 responses (response rate = 80%). To ensure data quality, we embedded two attention-check items and excluded questionnaires with more than 20% missing data (n = 132) or responses completed in under two minutes (n = 175). We also screened for straight-lining patterns (n = 42). Confidentiality was maintained by anonymizing responses and storing data on a secure server.

### 3.3. Participants

A total of 891 participants were recruited from 30 universities using a combination of convenience and snowball sampling techniques through online platforms (e.g., university mailing lists and social media channels).

### 3.4. Measures

Social media addiction was assessed using an adapted version of the Social Media Disorder (SMD) Scale (van den Eijnden et al., 2016). This nine-item scale captures core addiction symptoms such as preoccupation, tolerance, withdrawal, and functional impairment. Items (e.g., “During the past year, have you regularly found that you can’t think of anything else but the moment that you will be able to use social media again?”) were rated on a 5-point Likert scale ranging from 1 (Strongly Disagree) to 5 (Strongly Agree). Higher scores indicate a greater level of addictive behavior. Our data manifested that the reliability of this scale is 0.88 (Cronbach’s alpha).

Social pain was measured using the instrument developed by Stangier et al. (2021). This newly validated measure assesses the emotional distress associated with perceived social exclusion or rejection. The scale comprises 10 items (e.g., statements such as “When an acquaintance does not respond to me when I say hello, I feel rejected.”) rated on a 5-point Likert scale ranging from 1 (Not at all) to 5 (Extremely), with higher scores reflecting greater social pain. Previous research has demonstrated robust psychometric properties for this instrument and its internal consistency is quite high (Cronbach’s alpha = 0.87).

To measure trolling behaviors, we employed an updated and expanded version by (Sest & March, 2017) of the Global Assessment of Internet Trolling (GAIT) (Buckels et al., 2014). This measure comprises eight items. Participants responded using a 5-point Likert scale (1 = Strongly Disagree to 5 = Strongly Agree). For example, one item states, “Although some people think my posts/comments are offensive, I think they are funny”. The GAIT-Revised showed strong internal consistency (Cronbach’s alpha = 0.85), with higher scores indicating a greater propensity to engage in trolling behaviors

To assess college students’ language attitude, we administered the Chinese College Students’ Attitudes Toward Sexual Swear Words Scale (Wei & Chen, 2021). This scale comprises 49 items organized into three subscales: Cognition (17 items), Affection (17 items), and Behavior Tendency (15 items): the Cognition subscale evaluates students’ perceptions regarding the negative, insulting, and vulgar qualities of sexual swear words; the Affection subscale captures their emotional responses (e.g., feelings of discomfort or disgust); and the Behavior Tendency subscale measures the likelihood of using sexual swear words in various contexts (e.g., in public, in private, online). All items are rated on a 5-point Likert scale (1 = Strongly Disagree to 5 = Strongly Agree), with higher scores indicating a stronger negative attitude toward sexual swear words. The scale demonstrated strong psychometric properties, with Cronbach’s α coefficients ranging from 0.87 to 0.95, and evidence of good construct and criterion-related validity. In this study, only the subscale for Behavior Tendency is selected to measure language attitude and its reliability is also quite high (Cronbach’s alpha = 0.89).

### 3.5. Data Analysis

Data were analyzed using R (version 4.2.2) with the lavaan package (version 0.6-16) for structural equation modeling. Initially, data were screened for missing values, and distributions were examined to assess normality. Reliability for all scales was evaluated using Cronbach’s alpha. The measurement model was first established to confirm that the latent constructs were adequately represented by their indicators. As the moderating variable, language attitude, is a continuous variable, the moderation effects were tested via a SEM approach, mainly by dividing participants into three groups (high, medium, and low language attitude) based on their language attitude scores. Model fit was assessed using several indices, including the chi-square statistic (χ^2^), Comparative Fit Index (CFI), Tucker–Lewis Index (TLI), Root Mean Square Error of Approximation (RMSEA), and Root Mean Square Residual (RMSR).

## 4. Results

### 4.1. Preliminary Analysis

The sample comprised both undergraduate students (n = 671) and graduate students (n = 220). Participants ranged in age from 17 to 25 years (M = 20.4, SD = 2.1). The gender distribution was 482 males (54.1%) and 409 females (45.9%). Informed consent was obtained from all participants prior to data collection in accordance with ethical guidelines.

In order to assess the psychometric properties of our measurement instruments, descriptive statistics and reliability indices were computed for each latent construct based on data from 891 college students. The descriptive statistics include the sample size (n), mean, standard deviation, median, and range of scores. In addition, we evaluated internal consistency using two reliability coefficients: Cronbach’s alpha and McDonald’s Omega. While Cronbach’s alpha is the traditional measure of reliability under the assumption of tau-equivalence, McDonald’s Omega provides a more robust estimate when item loadings differ (McNeish, 2018). Convergent validity was further examined via the Average Variance Extracted (AVE), which indicates the proportion of variance captured by the latent construct relative to the measurement error, and the Composite Reliability (CR), which reflects the overall reliability of the construct (see Table 1).

For Factor 1 (Social Media Addiction), assessed by nine items, the mean score was 26.94 with a standard deviation of 9.67 and a median of 27, and the observed scores ranged from 9 to 45. Reliability analysis yielded Cronbach’s α = 0.908 and Omega = 0.908, while the AVE was 0.523 and the CR was 0.908, indicating satisfactory convergent validity and internal consistency.

Factor 2 (Social Pain), measured with 10 items, produced a mean of 29.97 (SD = 10.59, median = 30) with a score range of 10 to 50. This construct demonstrated excellent reliability, with Cronbach’s α and Omega both equal to 0.913, an AVE of 0.512, and a CR of 0.913.

Factor 3 (Internet Trolling Behavior) comprises eight items and yielded a mean of 23.84 (SD = 9.99, median = 23) with scores ranging from 8 to 40. The reliability indices for Factor 3 were exemplary, as evidenced by Cronbach’s α and Omega of 0.978, an AVE of 0.845, and a CR of 0.978.

Finally, Factor 4 (Language Attitude), measured by 15 items, had a mean score of 45.08 with a standard deviation of 15.76 and a median of 45, and the scores ranged from 15 to 75. This factor also demonstrated high internal consistency, with both Cronbach’s α and Omega equal to 0.941, an AVE of 0.518, and a CR of 0.941.

Collectively, these findings confirm that the latent constructs are measured reliably and that each factor exhibits acceptable convergent validity, thus providing a robust foundation for the subsequent analyses.

### 4.2. Correlation, Reliability and Validity

Table 2 displays the intercorrelations among the latent constructs. Factor 1 (Social Media Addiction) exhibits a moderate correlation with Factor 2 (Social Pain) (r = 0.622) and a lower association with Factor 3 (Internet Trolling Behavior) (r = 0.386), with virtually no relationship to Factor 4 (Language Attitude) (r = 0.001). Similarly, Factor 2 (Social Pain) is moderately correlated with Factor 3 (Internet Trolling Behavior) (r = 0.436) and shows negligible correlation with Factor 4 (Language Attitude) (r = −0.001), while Factor 3 (Internet Trolling Behavior) and Factor 4 (Language Attitude) are only minimally related (r = 0.053). Using the Fornell–Larcker criterion, the square roots of the AVE for each construct—approximately 0.723 for Factor 1, 0.715 for Factor 2, 0.919 for Factor 3, and 0.720 for Factor 4—exceed the respective inter-construct correlations, thereby supporting the discriminant validity of the measurement model.

### 4.3. Fit Indices

To evaluate measurement invariance across the three language attitude groups—high (n = 290), medium (n = 300), and low (n = 301)—a multigroup SEM was estimated. Initially, a configural model was specified, followed by the imposition of equality constraints to form a metric model. The model comparison, as presented in Table 3, revealed that the additional constraints did not significantly deteriorate model fit (χ^2^ difference = 31.075 with 48 degrees of freedom, *p* = 0.972). Based on this nonsignificant difference, the metric model was retained for subsequent analyses. The overall fit of the chosen multigroup model was excellent, as indicated by a Comparative Fit Index (CFI) of 0.994, a Root Mean Square Error of Approximation (RMSEA) of 0.020, and a Standardized Root Mean Square Residual (SRMR) of 0.032, thereby confirming the adequacy of the model across groups.

### 4.4. Structural Model and Moderation

The structural model was evaluated separately for the three groups based on language attitude levels—Group 1 (high), Group 2 (low), and Group 3 (medium)—with significance determined at the 0.01 level. In Group 1 (high language attitude), the path from Factor 1 (Social Media Addiction) to Factor 3 (Internet Trolling Behavior) was statistically significant (β = 0.547, *p* < 0.01), as was the path from Factor 2 (Social Pain) to Factor 3 (β = 0.931, *p* < 0.01). By contrast, in Group 2 (low language attitude), neither of these regression paths reached significance at the 0.01 threshold, indicating that in this subgroup the effects of Social Media Addiction and Social Pain on Internet Trolling Behavior were attenuated. In Group 3 (medium language attitude), both predictors exerted significant effects on Internet Trolling Behavior; however, the magnitude of these relationships was notably lower than in Group 1, with the coefficient for Social Media Addiction at β = 0.296 and that for Social Pain at β = 0.678 (both *p* < 0.01), as are shown in Table 4.

Furthermore, the covariance between Factor 1 (Social Media Addiction) and Factor 2 (Social Pain) was consistently positive and statistically significant across all groups (see Table 4). This robust covariance suggests that the interrelationship between Social Media Addiction and Social Pain remains stable irrespective of the differing levels of Language Attitude, reinforcing the notion that the association between these constructs is not contingent on variations in language sensitivity. These results underscore the moderating role of Language Attitude on the structural paths leading to Internet Trolling Behavior while also highlighting the invariant association between Social Media Addiction and Social Pain across groups.

## 5. Discussion

The present study aimed to investigate the interplay among social media addiction, social pain, and internet trolling while examining the moderating influence of language attitude among college students. The results provide compelling evidence that language attitude significantly shapes the pathway from both social media addiction and social pain to internet trolling. In groups characterized by high and medium levels of language attitude, both predictors contributed significantly to trolling behavior—with the high language attitude group displaying notably stronger effects—whereas, in the low language attitude group, these effects were absent. These findings underscore the pivotal role that individual differences in language sensitivity play in translating problematic online behaviors into overt forms of aggression.

With regard to the first hypothesis, social pain, characterized by the emotional distress arising from perceived rejection, exclusion, or devaluation in social interactions, appears to be a significant predictor of aggressive online behavior. Our findings indicate that individuals who report higher levels of social pain are more prone to engage in internet trolling, suggesting that the emotional turmoil stemming from negative social experiences can manifest externally as disruptive and provocative digital behavior. The theoretical framework underlying this relationship draws on the notion that social pain is processed in a manner similar to physical pain, triggering responses that are both immediate and intense (Sullivan & de C Williams, 2025). When an individual experiences social pain, the resulting emotional distress may compromise their ability to regulate emotions effectively (Yu et al., 2024). This dysregulation can lead to maladaptive coping mechanisms, such as internet trolling, which serve as an outlet for the internal distress (Buckels et al., 2019). In this context, trolling may function as a compensatory behavior—a way for individuals to express their inner hurt by lashing out in online spaces where consequences are less immediate and personal (Kim et al., 2025).

Moreover, our results are consistent with previous literature that identifies social pain as a critical factor in the development of aggressive behaviors online (Kim et al., 2025; Kunstman et al., 2025; Merritt et al., 2025). The digital environment, characterized by anonymity and a lack of immediate accountability, offers a convenient venue for individuals to externalize their emotional pain (Avci et al., 2024). When people feel isolated or undervalued in their interpersonal relationships, they may resort to trolling as a method of reclaiming a sense of power or control (Bishop, 2015b). The significant relationship observed in our analysis underscores the idea that social pain is not just a passive state of suffering but an active force that can drive individuals toward behaviors intended to inflict emotional discomfort on others (Merritt et al., 2025; Sullivan & de C Williams, 2025).Trolling, in this light, can be viewed as a reactive process whereby individuals attempt to counterbalance their internal pain by provoking similar feelings in others, thereby establishing a temporary, albeit unhealthy, equilibrium (Buckels et al., 2019; Slavich et al., 2019).

As for the second hypothesis, in the analysis, individuals exhibiting higher levels of social media addiction were more likely to engage in trolling, particularly evident in groups characterized by heightened sensitivity toward language. In the high language attitude group, the regression analysis yielded a robust positive relationship (β = 0.547), while the medium language attitude group also demonstrated a significant, albeit less pronounced, effect (β = 0.296). These findings indicate that the compulsive engagement with social media platforms can predispose individuals to adopt aggressive and provocative online behaviors.

The mechanisms underlying this relationship may be rooted in the design features of social media platforms that promote addictive usage patterns (Abdulsalim et al., 2025; Kochukrishna Kurup et al., 2025; Yang et al., 2025; Yoosefi et al., 2025). Features such as endless scrolling, real-time notifications, and the intermittent reinforcement of likes and comments contribute to a loss of self-regulatory control. As users become increasingly absorbed in their digital interactions, the impulse control required to moderate responses in online environments may deteriorate. This diminished self-regulation can lower the threshold for engaging in impulsive, and sometimes aggressive, behaviors. In such a context, internet trolling emerges not merely as a form of attention-seeking but as an extension of the compulsive and dysregulated patterns fostered by social media addiction (Liao et al., 2025; Lorenzo-Rumbo et al., 2025; Oral & Karakurt, 2025).

Theoretically, these findings extend our understanding of the behavioral consequences of social media addiction by linking it to antisocial online behaviors like trolling. Practically, the results underscore the need for targeted interventions that not only address addictive online behaviors but also bolster self-regulation and digital literacy (Aksoy et al., 2025; Feng et al., 2025; Yao et al., 2025). By developing strategies to mitigate compulsive social media use, and by fostering a more reflective approach to online language and interaction, it may be possible to reduce the incidence of trolling. This, in turn, could contribute to creating a healthier digital communication environment, where the negative impacts of excessive social media engagement are minimized.

Focusing on the third hypothesis, our findings offer robust support for the proposition that social media addiction is strongly positively correlated with social pain. Social media addiction is characterized by a compulsive preoccupation with digital interactions, a constant need for online validation, and a loss of control over one’s social media engagement. This addictive behavior not only disrupts daily functioning but also seems to intensify feelings of emotional distress that are central to social pain. Individuals who find themselves entrapped in the cycle of excessive social media use are more likely to experience a deep sense of isolation, rejection, and inadequacy, even in the midst of constant online connectivity.

One explanation for this relationship lies in the inherently dual-edged nature of social media platforms. Although these platforms are designed to facilitate connections and provide avenues for social support, they simultaneously create environments rife with social comparison (Jahagirdar et al., 2024; Saqib & Gazerani, 2024; Zeng et al., 2024). Users are constantly exposed to idealized images and curated portrayals of others’ lives, which can lead to a phenomenon often described as “compare and despair”. When individuals compare their everyday experiences with the seemingly flawless lives presented online, they may feel inferior or excluded, thus heightening their social pain (Huang et al., 2025; Kalınkara & Talan, 2025). The compulsive nature of social media addiction ensures that users are continuously engaging with such content, reinforcing a cycle where the more they use these platforms, the more acute their feelings of social isolation and rejection become.

Additionally, the pervasive nature of social media addiction often results in a significant reduction in face-to-face interactions, which are crucial for developing deep, meaningful relationships. As individuals become increasingly absorbed in their online interactions, they may neglect real-life social engagements that provide genuine emotional support and validation. This shift away from in-person contact can leave individuals with a diminished sense of belonging and community, further exacerbating their experience of social pain. In essence, while social media addiction might offer a temporary escape or a sense of connection, it ultimately undermines the quality of real-world interactions, thereby contributing to persistent feelings of loneliness and emotional distress (Sao et al., 2024).

In terms of the fourth hypothesis, social pain, and internet trolling, the results of this study highlight the crucial role that individual differences in language evaluation play in shaping online behavior. Language attitude, defined here as the evaluative stance individuals hold toward offensive language, appears to influence how internal distress and compulsive digital engagement translate into overt aggressive actions. Our analysis revealed that the associations between both social media addiction and social pain with internet trolling were significantly stronger among individuals with a high negative language attitude, while these relationships were markedly attenuated or non-significant in individuals with a lower negative language attitude.

For those with a high negative language attitude, the sensitivity to language nuances intensifies the reaction to provocative or offensive content online. In such individuals, even minor instances of perceived insult or derogatory language can trigger a disproportionate emotional response. This heightened sensitivity appears to lower the threshold for engaging in aggressive behaviors, such as internet trolling, as a means of expressing frustration or reclaiming a sense of control. In effect, social media addiction and social pain act as catalysts that are amplified by an adverse language attitude, leading to a greater likelihood of engaging in disruptive online behavior. The moderating effect observed suggests that language attitude does not merely coexist with these phenomena—it actively shapes the pathway from emotional and behavioral precursors to tangible online aggression.

Conversely, among individuals with a low negative language attitude, the link between internal states—whether stemming from addictive patterns of social media use or from the experience of social pain—and external manifestations like trolling is much weaker. In these individuals, a more tolerant or neutral stance toward offensive language seems to provide a protective buffer that dampens the impulse to translate internal distress into external aggression. This finding implies that the cognitive appraisal of language cues plays a pivotal role in determining whether negative emotions will be expressed in hostile online interactions.

A notable contribution of this study is its pioneering integration of language attitude as a moderating factor in explaining how social pain and social media addiction culminate in internet trolling. Traditionally, research on online aggression has centered on the direct effects of emotional distress and compulsive digital behavior, often neglecting the nuanced role that language attitude or specific language play in shaping these outcomes. By introducing language attitude into the model, this work breaks new ground and demonstrates that the way individuals perceive and react to offensive language is not merely a peripheral trait but a critical determinant that can intensify or mitigate the drive to troll. Moreover, our findings reaffirm that language attitude encompasses a distinct behavioral dimension—a behavior tendency that influences how one engages in online interactions (Ajzen & Fishbein, 1980; Halpin et al., 2024). In this regard, individuals with a heightened negative language attitude are not simply holding a cognitive evaluation; they are predisposed to act on that evaluation, which amplifies the likelihood of translating internal distress into overt trolling behavior (Ajzen & Madden, 1986). This dual function of language attitude—as both a cognitive filter and an active behavioral catalyst—provides deeper insight into the dynamics of online aggression. Ultimately, this study not only elucidates the interplay among social pain, social media addiction, and internet trolling but also reinstates the importance of recognizing language attitude as an essential behavioral factor. This perspective opens new avenues for developing targeted interventions that address not just the symptoms of digital overuse and emotional distress but also the underlying linguistic sensitivities that propel aggressive online conduct.

It should be noted that the language–attitude scale employed in the present study focuses exclusively on a single prototypical form of offensive language—sexual swear words (Wei & Chen, 2021)—whereas, in everyday discourse, language attitudes are also shaped by exposure to racial, religious, or other generalized slurs. This limitation stems from two considerations. First, the sample’s racial and religious composition was relatively homogeneous, precluding the inclusion of items targeting those categories of derogatory language. Second, within the linguistic environment experienced by our participants, sexual swear words are particularly frequent and thus provided a more representative basis for measurement. As is shown in a survey, 71% of the sampled students use sexual swear words on a daily basis (Wei & Chen, 2021). Nonetheless, this design remains congruent with Soukup’s (2012) conceptualization of language attitude as a structured, relatively stable internal state comprising three components: cognition, affect, and behavioral tendency. Future researchers might draw on this definition to develop dedicated scales for racial and religious slurs, select suitably diverse samples, and investigate the broader, multidimensional effects of language attitude—thereby enhancing the generalizability and ecological validity of subsequent findings.

## 6. Conclusions, Implications, Limitations and Future Directions

In summary, the current study demonstrates that language attitude plays a critical moderating role in the relationship between social media addiction, social pain, and internet trolling. The findings highlight the need for a more nuanced approach in both theoretical modeling and practical interventions aimed at mitigating the adverse effects of problematic social media use. Addressing language attitudes may represent a promising avenue for reducing the incidence of online trolling and fostering healthier digital communication among college students.

Theoretically, these findings extend our understanding of online antisocial behaviors by integrating language attitude into the established framework linking social media addiction and social pain to internet trolling. They suggest that language attitude is not merely a peripheral or passive trait, but an active moderator that can intensify or mitigate the behavioral consequences of digital overuse and emotional distress. Practically, this insight has significant implications for intervention strategies. For instance, programs aimed at reducing online aggression might benefit from incorporating components that address individuals’ sensitivity to offensive language. By moderating language attitudes—perhaps through media literacy or cognitive-behavioral techniques—practitioners may be able to curb the progression from social media addiction and social pain to destructive trolling behaviors.

Despite its contributions, the study is not without limitations. The cross-sectional design precludes definitive causal interpretations, and the reliance on self-report measures may introduce response biases. Structural equation modeling (SEM) as a research paradigm is fundamentally grounded in the extraction of intervariable associations from the variance–covariance matrix and thus does not possess intrinsic causal-inference properties. Consequently, researchers who wish to explore treatment effects from a causal standpoint must first identify and account for potential confounding variables—such as personality traits and cultural factors—and explicitly specify the causal pathways of interest. Moreover, they should adopt methodological frameworks capable of isolating causal impacts (e.g., instrumental-variable approaches or difference-in-differences designs) in order to achieve more precise and valid conclusions. Furthermore, given that the sample consisted exclusively of college students, the generalizability of the findings to other age groups or cultural contexts remains uncertain. Future research would benefit from longitudinal designs and more diverse samples, as well as the exploration of additional moderating factors such as personality traits or social context, which could further clarify the mechanisms underpinning online antisocial behavior.

## Figures and Tables

**Table 1:** Descriptive Statistics of the Collected Data.

	Mean	sd	Range	Item Amout	Item Mean	Alpha	Omega	AVE	CR
Social Media Addiction	26.935	9.671	36.000	9.000	2.993	0.908	0.908	0.523	0.908
Social Pain	29.973	10.589	40.000	10.000	2.997	0.913	0.913	0.512	0.913
Internet Trolling	23.838	9.993	32.000	8.000	2.980	0.978	0.978	0.845	0.978
Language Attitude	45.082	15.755	60.000	15.000	3.005	0.941	0.941	0.518	0.941

**Table 2:** Correlation Matrix Among Factors.

	Social Media Addiction	Social Pain	Internet Trolling	Language Attitude
Social Media Addiction	1.000			
Social Pain	0.622	1.000		
Internet Trolling	0.386	0.436	1.000	
Language Attitude	0.001	−0.001	0.053	1.000

**Table 3:** Model Comparison.

	Df	AIC	BIC	Chisq	Chisq.diff	Df.diff	Pr.Chisq.
Configural Model	963	65,453.845	66,273.336	1081.683			
Metric Model	1011	65,379.278	65,968.736	1103.116	31.075	48	0.972

**Table 4:** Structural Model Results.

lhs	op	rhs	Group	est	S.E.	z	*p*
**Internet Trolling**	**~**	**Social Media Addiction**	**High**	**0.564**	**(0.078)**	**7.219**	**<0.001**
**Internet Trolling**	**~**	**Social Pain**	**High**	**0.728**	**(0.088)**	**8.304**	**<0.001**
**Social Media Addiction**	**~~**	**Social Pain**	**High**	**0.685**	**(0.084)**	**8.139**	**<0.001**
Social Media Addiction	~~	Social Media Addiction	High	1.123	(0.114)	9.876	<0.001
Social Pain	~~	Social Pain	High	1.018	(0.127)	8.016	<0.001
Internet Trolling	~~	Internet Trolling	High	0.635	(0.064)	9.888	<0.001
**Internet Trolling**	**~**	**Social Media Addiction**	**Low**	**–0.177**	**(0.082)**	**–2.167**	**0.030**
**Internet Trolling**	**~**	**Social Pain**	**Low**	**–0.042**	**(0.085)**	**–0.488**	**0.626**
**Social Media Addiction**	**~~**	**Social Pain**	**Low**	**0.642**	**(0.076)**	**8.397**	**<0.001**
Social Media Addiction	~~	Social Media Addiction	Low	0.971	(0.111)	8.741	<0.001
Social Pain	~~	Social Pain	Low	0.993	(0.121)	8.223	<0.001
Internet Trolling	~~	Internet Trolling	Low	0.983	(0.085)	11.606	<0.001
**Internet Trolling**	**~**	**Social Media Addiction**	**Medium**	**0.264**	**(0.103)**	**2.570**	**0.010**
**Internet Trolling**	**~**	**Social Pain**	**Medium**	**0.562**	**(0.110)**	**5.116**	**<0.001**
**Social Media Addiction**	**~~**	**Social Pain**	**Medium**	**0.734**	**(0.081)**	**9.113**	**<0.001**
Social Media Addiction	~~	Social Media Addiction	Medium	1.010	(0.118)	8.560	<0.001
Social Pain	~~	Social Pain	Medium	0.952	(0.115)	8.276	<0.001
Internet Trolling	~~	Internet Trolling	Medium	0.808	(0.069)	11.705	<0.001

Notes: in the op column, ~ means regression and ~~ means variance covariance.

## Data Availability

The data presented in this study are available upon request from the corresponding author. The data are not publicly available because they consist of raw data that have been processed and analyzed for the purposes of this study.
